# Risk of cardiovascular disease and mortality in patients with diabetes and acute pancreatitis history: a nationwide cohort study

**DOI:** 10.1038/s41598-022-21852-7

**Published:** 2022-11-04

**Authors:** Dong Kee Jang, Jin Ho Choi, Woo Hyun Paik, Ji Kon Ryu, Yong-Tae Kim, Kyung-Do Han, Sang Hyub Lee

**Affiliations:** 1 Department of Internal Medicine, Seoul Metropolitan Government Boramae Medical Center, Seoul National University College of Medicine, Seoul, Korea; 2 Department of Internal Medicine and Liver Research Institute, Seoul National University Hospital, Seoul National University College of Medicine, 101 Daehak-ro, Jongno-gu, Seoul, 03080 Korea; 3 Department of Statistics and Actuarial Science, Soongsil University, 369 Sangdo-ro, Dongjak-gu, Seoul, 06978 Korea

**Keywords:** Cardiology, Endocrinology, Gastroenterology

## Abstract

Patients with acute pancreatitis (AP) may have an increased risk of cardiovascular disease (CVD). Few studies have dealt with the association between AP and the risk of CVD in diabetic patients. This study aimed to investigate the risk of CVD and mortality in patients with diabetes and AP history by analyzing a large-scale national claims database in Korea. Data from the Korean National Health Insurance Service database was analyzed. A total of 2,746,988 participants with type 2 diabetes mellitus that underwent a general health examination between 2009 and 2012 were enrolled. The participants were divided into two groups according to AP history (yes or no) prior to the examination date, and follow-up data until 2018 was analyzed. The primary endpoint was the occurrence of stroke, myocardial infarction (MI), or death. The Cox proportional hazards regression analysis was used to evaluate the association between AP history and the risk of stroke, MI, and mortality. After exclusion, the included number of participants with and without AP history were 3,810 and 2,258,910, respectively. The presence of AP history showed a significantly higher incidence of stroke, MI, and mortality. The adjusted hazard ratios (95% confidence interval) for the risk of stroke, MI, and mortality were 1.534 (1.342–1.753), 1.998 (1.733–2.303), and 2.353 (2.200–2.515), respectively. Age < 65, male sex, current smoking, and drinking significantly increased the risk of death in the subgroup analyses. The risk of stroke, MI, and mortality was significantly higher in diabetic participants with AP history than those without AP history at 9-year follow-up. This suggests that active management of cardiovascular risk factors is necessary in diabetic patients with AP history.

## Introduction

Acute pancreatitis (AP) is a leading cause of inpatient care among gastrointestinal diseases in the United States with a substantial economic burden (> $2.6 billion per year)^1^. The incidence of AP ranges from 5 to 30 cases per 100,000 which has been rising in recent years^2,3^. Although AP itself has a high mortality rate in severe cases^4^, recent studies have shown that patients with AP also have an increased risk (adjusted hazard ratio [HR], 1.24–1.76) of cardiovascular disease (CVD) compared to controls^5,6^. Inflammatory cytokines, hemodynamic disturbances, and metabolic disorders related to AP may play an important role in increasing the risk of CVD^5^. On the contrary, there is also a report that CVD may heighten the risk of AP^7^, suggesting a close association between CVD and AP in the short and long term. However, further research is still needed to suggest the exact mechanism.

CVD is recognized as the leading cause of death in the world which accounts for 32% of deaths^8^. The majority of CVD-related deaths are attributable to either coronary heart disease or cerebrovascular disease. AP is considered to affect long-term morbidity and mortality by increasing the risk of CVD, and also influence short-term mortality due to problems of the pancreatitis itself. It is essential to consider well-known risk factors in the process of estimating the risk of CVD. In particular, diabetes is the most important independent risk factor for the occurrence of CVD^9^. The American Diabetes Association recommends the systematic and annual assessment of CVD risk factors in all patients with diabetes. Such risk factors include obesity, hypertension, dyslipidemia, smoking, history of premature coronary heart disease, and presence of albuminuria^9^. Efforts to modify such risk factors were actually known to significantly lower long-term mortality and morbidity in diabetic patients^10^.

As mentioned above, while AP was regarded to elevate the risk of CVD, previous studies did not consider important factors such as smoking^5,6^. Furthermore, few studies have dealt with the association between AP and the risk of CVD in diabetic patients. In this study, we aimed to investigate the risk of CVD and mortality in patients with diabetes and AP history by analyzing a large-scale national representative claims database in Korea.

## Methods

### Dataset

Data from the Korean National Health Insurance Service (NHIS) database was analyzed. The NHIS, a single insurer in Korea, covers about 97% of the total population, and its database includes demographic information, medical treatment claims, general health examination results, and death information. The claims-based database includes records of diagnoses, prescriptions, hospitalizations, and level of institutions, all of which was provided after deidentification. The general health examination is comprised of a health history questionnaire, measurements of anthropometric index and blood pressure, blood sampling after an overnight fast, urinalysis, and chest X-ray. The International Statistical Classification of Diseases and Related Health Problems 10th revision (ICD-10) codes were used for diagnosis. The study protocol was approved by The Soongsil University Institutional Review Board (No. SSU-202003-HR-201-01). The need for written informed consent was waived by decision of the Soongsil University Institutional Review Board. All methods were carried out in accordance with relevant guidelines and regulations.

### Study population

A total of 2,746,988 participants with type 2 diabetes mellitus (T2DM) that underwent a general health examination between 2009 and 2012 were retrospectively enrolled. Participants under the age of 20, those who had been diagnosed with a stroke or myocardial infarction (MI) before and within one year following the examination date (lag period), and those with missing data were excluded from the study. The remaining 2,264,074 participants were divided into two groups according to AP history (yes or no) within three years prior to the examination date. The follow-up records of these participants through 2018 were collected and analyzed. New cases of AP in participants who had no previous history of AP after the examination date were excluded from the analysis. Participants of national health examinations provided written informed consent for the use of their data for research. Only unidentified data were used for analysis.

### Definitions

All diagnoses were determined by combining ICD-10 codes and operational definitions. T2DM diagnosis was defined as follows: (1) The presence of ICD-10 codes E11-E14 and claims for at least one oral anti-diabetic agent or insulin at the baseline, or (2) general health examination results of fasting glucose level ≥ 126 mg/dL. AP was defined as the presence of code K85 and hospitalization during the same period.

The primary endpoint of this study was the occurrence of stroke, MI, or death. Since the endpoint was considered to have been reached if any of stroke, MI, or death occurred, deaths from stroke or MI were not included into the endpoints. Stroke was defined by I63 or I64 codes, with a history of hospitalization plus claims for brain imaging (magnetic resonance imaging or computed tomography). MI was defined as hospitalization with I21 or I22 codes. Hypertension was defined by I10-I13 and I15 codes or a systolic/diastolic blood pressure ≥ 140/90 mmHg, with at least one claim for the prescription of anti-hypertensive agents. Dyslipidemia was defined as the presence of at least one claim per year for anti-hyperlipidemic agents with code E78 or total cholesterol ≥ 240 mg/dL. Regular exercise was defined as > 30 min of moderate physical activity at least 5 times/week or > 20 min of strenuous physical activity at least 3 times/week.

### Statistical analysis

Baseline characteristics are presented as number (%), mean ± standard deviation, or geometric means (95% confidence interval [CI]). Variables between the two groups were compared using Student’s t-test for continuous variables, and the Pearson’s chi-square test for categorical variables. The Cox proportional hazards regression analysis was used to evaluate the association between AP history and the risk of stroke, MI, and death. During analyses, five models were fitted to gradually reduce confounding associations. Model 1 was an unadjusted model. Model 2 was adjusted for age and sex. Smoking, alcohol drinking, regular exercise, and income level were added sequentially after age and sex in Model 3. Comorbidities (hypertension and dyslipidemia) and body mass index (BMI) were added to Model 4, and diabetes-related variables (diabetes duration, use of insulin, and prescription of two or more oral diabetic agents) were further added to Model 5. Subgroup analysis was performed based on Model 5 with seven binary variables (age 65, sex, current smoking, alcohol drinking, hypertension, dyslipidemia, and BMI 25 kg/m^2^). These results were expressed as HRs with 95% CI. The cumulative incidence probabilities of stroke, MI, and mortality were plotted using Kaplan–Meier curves. Statistical significance was defined as two-sided *p* value < 0.05. All statistical analyses were performed using SAS version 9.3 (SAS Institute Inc., Cary, NC, USA) and R version 3.2.3 (The R Foundation for Statistical Computing, Vienna, Austria; http://www.Rproject.org).

### Ethics approval and consent to participate

The study protocol was approved by The Soongsil University Institutional Review Board. The need for written informed consent was waived.

## Results

### Baseline characteristics

The number of participants with and without AP history were 3,810 and 2,258,910, respectively. As shown in Table 1, the group with AP history had significantly lower age, body mass index, waist circumference, renal function, blood pressure, and cholesterol levels compared to the group without AP history (control group). On the contrary, proportions of male sex, use of insulin, intake of two or more anti-diabetic agents, current smokers, heavy drinkers, low-income participants, and levels of liver-related enzymes were significantly higher in the group with AP history. However, the proportion of participants with hypertension, dyslipidemia, long-standing diabetes, and those doing regular exercise were not significantly different between the two groups.

**Table 1:** Baseline characteristics according to acute pancreatitis history.

Variables	AP history		*p* value
	No (n = 2,258,910)	Yes (n = 3,810)	
Age, yr	55.65 ± 12.32	54.97 ± 11.49	0.0007
Male sex	1,383,936 (61.27)	3,086 (81.00)	< .0001
BMI, kg/m^2^	25.07 ± 3.69	23.61 ± 3.61	< .0001
Hypertension	1,172,968 (51.93)	1,972 (51.76)	0.836
Dyslipidemia	849,239 (37.60)	1,469 (38.56)	0.2209
Treatment for diabetes ≥ 5 yr	618,323 (27.37)	1,039 (27.27)	0.8875
Use of insulin	165,928 (7.35)	1,274 (33.44)	< .0001
Oral antidiabetic agent ≥ 2	830,318 (36.76)	1,778 (46.67)	< .0001
**Smoking status**			< .0001
Never	1,225,400 (54.25)	1,259 (33.04)	
Ex-smoker	403,544 (17.86)	637 (16.72)	
Current smoker	629,966 (27.89)	1,914 (50.24)	
**Drinking status, g/day**			< .0001
None	1,231,754 (54.53)	1,849 (48.53)	
Mild (< 30)	783,623 (34.69)	1,239 (32.52)	
Heavy (> 30)	243,533 (10.78)	722 (18.95)	
Income lower, 20%	432,670 (19.15)	916 (24.04)	< .0001
Regular exercise	468,443 (20.74)	815 (21.39)	0.3202
Waist circumference, cm	85.21 ± 8.85	83.51 ± 8.69	< .0001
GFR, mL/min	85.89 ± 36.35	89.41 ± 28.01	< .0001
SBP, mmHg	128.73 ± 15.79	126.4 ± 16.20	< .0001
DBP, mmHg	79.17 ± 10.27	78.54 ± 10.77	0.0001
Total cholesterol, mg/dL	198.55 ± 46.36	192.14 ± 54.47	< .0001
HDL-C, mg/dL	51.44 ± 13.48	51.84 ± 16.77	0.0685
LDL-C, mg/dL	113.84 ± 88.10	100.61 ± 58.67	< .0001
AST, IU/L	26.19 (26.18–26.21)	31.41 (30.8–32.04)	< .0001
ALT, IU/L	26.56 (26.54–26.58)	27.38 (26.8–27.96)	0.0012
GGT, IU/L	37.17 (37.14–37.21)	61.58 (59.5–63.74)	< .0001
Triglyceride, mg/dL	145.95 (145.84–146.06)	158.38 (155.02–161.81)	< .0001

Values are presented as number (%), mean ± standard deviation, or geometric means (95% confidence interval).

*AP* acute pancreatitis; *BMI* body mass index; *GFR* glomerular filtration rate; *SBP* systolic blood pressure; *DBP* diastolic blood pressure; *HDL-C* high density lipoprotein cholesterol; *LDL-C* low density lipoprotein cholesterol; *AST* aspartate aminotransferase; *ALT* alanine aminotransferase; *GGT* gamma-glutamyl transferase.

### Risk of cardiovascular events and mortality according to history of acute pancreatitis

**Figure 1:**
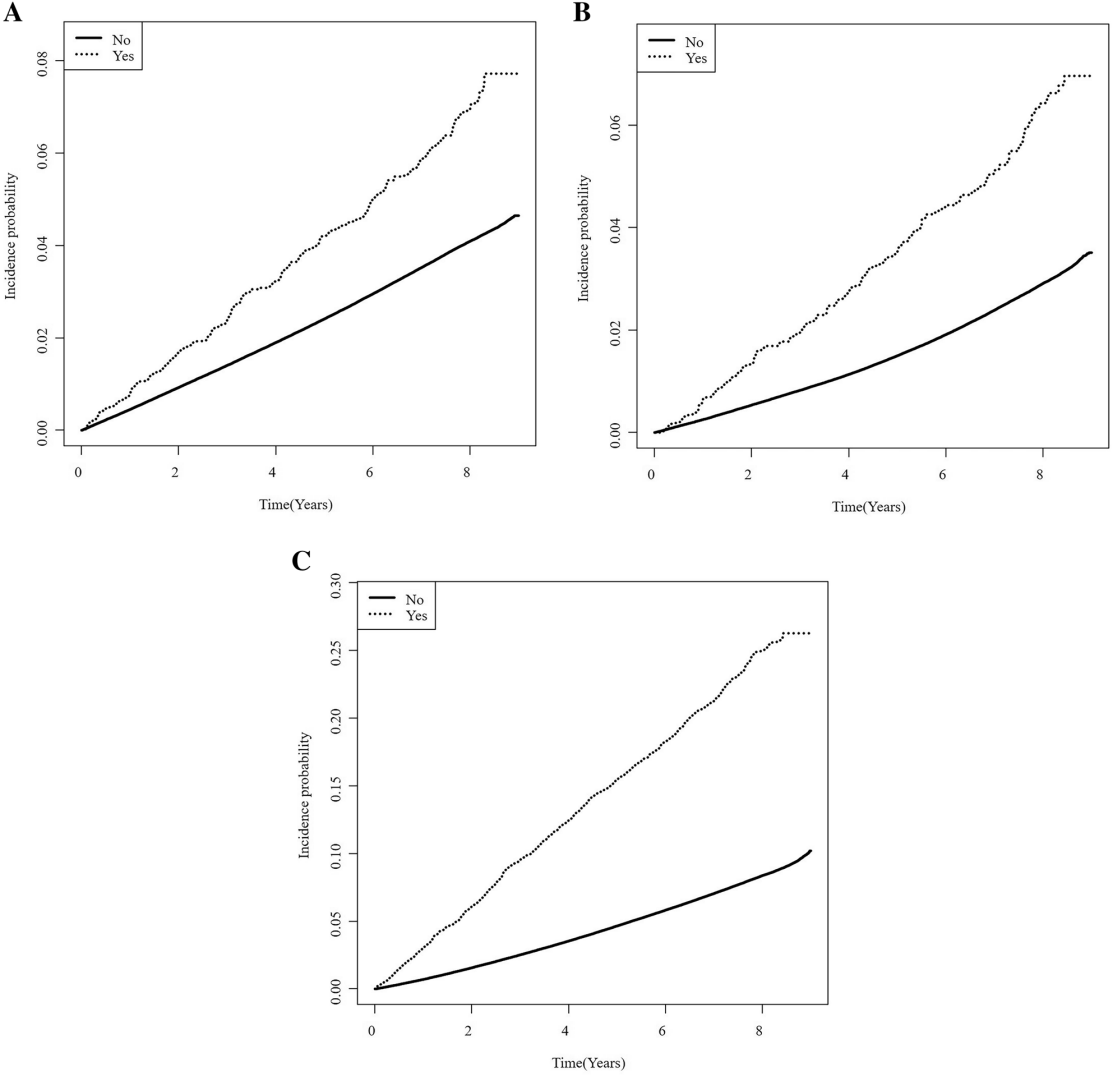
Kaplan–Meier curves for the risk of cardiovascular disease and mortality according to acute pancreatitis history. The curves display the cumulative incidence probability of (**A**) stroke, (**B**) myocardial infarction, and (**C**) mortality.

Table 2 displays the results of the Cox proportional hazard regression analysis evaluating the independent risk of stroke, MI, and mortality according to AP history. The presence of AP history shows a significantly higher incidence of stroke, MI, and mortality. The Kaplan–Meier curves for the incidence probability of each event during the follow-up period are provided in Fig. 1. All log-rank *p* values were < 0.0001. The crude HRs (95% CI) for the risk of stroke, MI, and mortality were 1.858 (1.626–2.123), 2.549 (2.212–2.937), and 3.500 (3.274–3.741), respectively. After adjusting for age and sex (Model 2), the HRs were slightly decreased except for stroke. Adjusting for social factors (Model 3) and comorbidities (Model 4), the HRs of all events were further decreased, and the HR of mortality was decreased the most. After the diabetes-related variables were adjusted in the final model, it is noted that the HRs of all events were reduced the most overall. In the final model (Model 5), it is confirmed that AP history still increases the risk of stroke, MI, and mortality by 1.5, 2.0, and 2.4-fold compared to controls, respectively.

**Table 2:** Risk of stroke, myocardial infarction, and mortality according to acute pancreatitis history.

AP history	N	Event	Incidence rate, /1,000 person-yr	Hazard ratio (95% confidence interval)				
				Model 1	Model 2	Model 3	Model 4	Model 5
		Stroke						
No	2,067,549	72,092	4.82	1 (Ref.)	1 (Ref.)	1 (Ref.)	1 (Ref.)	1 (Ref.)
Yes	3,810	217	8.90	1.858 (1.626,2.123)	1.858 (1.626,2.122)	1.748 (1.53,1.997)	1.731 (1.515,1.978)	1.534 (1.342,1.753)
		MI						
No	2,067,549	47,377	3.15	1 (Ref.)	1 (Ref.)	1 (Ref.)	1 (Ref.)	1 (Ref.)
Yes	3,810	192	7.86	2.549 (2.212,2.937)	2.495 (2.165,2.875)	2.304 (1.999,2.655)	2.287 (1.984,2.635)	1.998 (1.733,2.303)
		Mortality						
No	2,067,549	153,400	10.11	1 (Ref.)	1 (Ref.)	1 (Ref.)	1 (Ref.)	1 (Ref.)
Yes	3,810	871	34.88	3.500 (3.274,3.741)	3.335 (3.12,3.564)	3.087 (2.888,3.299)	2.860 (2.676,3.057)	2.353 (2.200,2.515)

Model 1, unadjusted; Model 2, adjusted for age and sex; Model 3, adjusted for model 2 + smoking, alcohol drinking, regular exercise, and income level; Model 4, adjusted for model 3 + hypertension, dyslipidemia, and body mass index; Model 5, adjusted for model 4 + diabetes duration, use of insulin, and prescription of two or more oral diabetic agents.

*AP* acute pancreatitis; *MI*, myocardial infarction.

### Risk of cardiovascular events and mortality in subgroups

**Figure 2:**
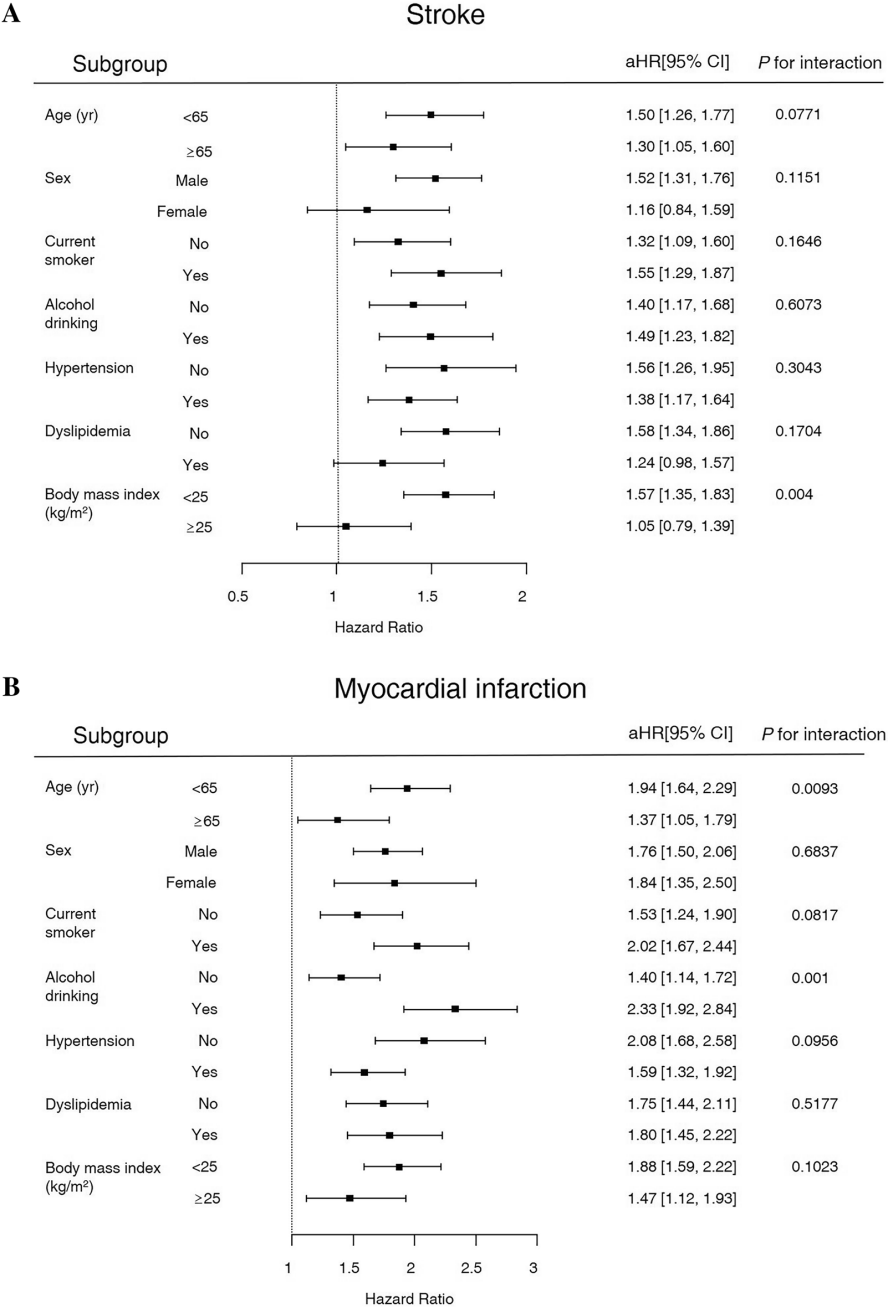

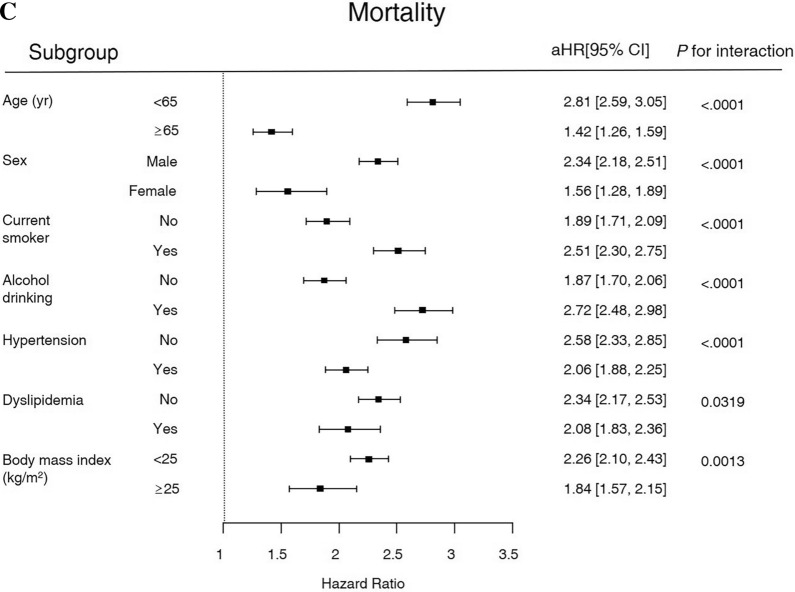
Forest plots for the risk of cardiovascular disease and mortality in subgroup analyses. (**A**) HR of stroke, (**B**) HR of myocardial infarction, and (**C**) HR of mortality.

The results of subgroup analysis regarding the risk of stroke, MI, and mortality by age, sex, smoking status, drinking status, hypertension, dyslipidemia, and BMI are presented in Fig. 2. Among participants with AP history, the risk of stroke was significantly higher in those with BMI < 25 kg/m^2^. The risk of MI was significantly higher in those < 65 yr and drinking. In terms of mortality, age < 65 yr, male sex, current smoking, and drinking significantly increased the risk of death. In addition, low BMI, and absence of hypertension or dyslipidemia increased mortality in diabetic participants with AP history.

## Discussion

In this large-scale nationwide cohort study, the independent risk of stroke, MI, and mortality was significantly higher in diabetic participants with AP history in comparison to those without AP history at the 10-year follow-up. The adjusted HRs were 1.5–2.4 after considering important confounding variables. The results of this study suggest that active management of cardiovascular risk factors is necessary in diabetic patients with AP history.

A recent similar large-scale cohort study reported that the adjusted HR of acute CVD was 1.76 (95% CI, 1.47–2.12) in patients with AP which is comparable to our result^5^. However, unlike our study, the report did not adjust important confounding factors such as smoking and alcohol. In addition, the report did not consider the time sequence of AP occurrence and CVD during the research period, making it difficult to assess causality. In contrast, our study displays a possible causality as we constructed a separate cohort of diabetic patients with AP history but no CVD, and also conducted a follow-up to evaluate the CVD incidence or death for a long period of time. Another similar cohort study suggested that the risk of acute coronary syndrome is higher in patients with first AP than those without AP (adjusted HR 2.46, in diabetic patients)^6^. The study also showed that approximately one third of acute coronary syndrome developed within one month of AP occurrence. However, the study did not include a lag period which implies the inclusion possibility of many CVD patients before AP. Moreover, the study also did not adjust for important factors such as smoking or BMI.

AP is an acute systemic inflammatory process that is associated with the variable involvement of one or more organ systems in varying degrees^11^. In particular, the cardiovascular system may be affected in all stages of AP which includes hemodynamic, cardiac rhythm, and pericardial changes. Therefore, fluid resuscitation is important in the early stages of AP^12–14^. AP is also known to be associated with disturbances in microcirculation^15^. It is assumed that such changes play an important role in cardiovascular outcomes in the short term, and may affect the occurrence of CVD through atherosclerosis in the long term^5^. The release of several inflammatory cytokines such as tumor necrosis factor α, interleukin (IL)-6, IL-10, and the chemokine monocyte chemoattractant protein-1 in the process of AP^16^ may be associated with the pathogenesis of atherosclerosis^17,18^. However, more research is needed to elucidate the exact mechanism.

As shown in Table 1, insulin or additional anti-diabetic agents are often required for diabetic patients with AP history. This suggests that AP might be associated with the level of endocrine dysfunction, which appears to affect the severity of diabetes, which in turn might increase the CVD risk. A previous meta-analysis showed that prediabetes and/or DM was observed in 37% individuals after AP, with a relative risk of 2.7 at 5 years after diagnosis^19^. Accordingly, a large portion of diabetic participants with AP history might belong to pancreatogenic, or type 3c, diabetes^20^. However, it is difficult to confirm the causal relationship between AP and diabetes in our study, as we identified participants with diabetes who had a history of AP at the baseline.

The Cox proportional hazard regression analysis showed the highest HR to be all-cause mortality (2.353), followed by in MI (1.998) and stroke (1.534) from Model 5 (Table 2). The simple summation of incidence rate per 1000-person-year for CVD was 16.76 and that of all-cause mortality was 34.88 in participants with AP history. Therefore, it is expected that other factors contributing to mortality other than CVD clearly exist. Although we have not been able to investigate the cause of death in each patient, there may have been patients who died from various malignant neoplasms known to increase with diabetes, particularly the increased risk of pancreatic cancer following AP diagnosis^21^. Also, there may have been patients who died from other fatal diabetic complications such as infection or end stage renal disease^22^. Interestingly, subgroup analysis showed that the mortality was higher in younger participants (< 65 yr) with no hypertension or dyslipidemia (Fig. 2). Combining such results, it was noted that the long-term prognosis was worse when AP occurred at a younger age. In such cases, conditions other than CVD may contribute significantly to the mortality rate. Progression to chronic pancreatitis (CP) from AP is more likely for drinkers who experience initial AP event at a younger age^23^. It is known that the mortality rate is higher when AP progresses to CP^24^, and thus several factors associated with the AP-CP sequence may have greatly contributed to the increase in mortality among younger participants in our study. However, as the occurrence of CP was not evaluated as an outcome in our study, it was difficult to determine the substantial impact of CP.

In fact, risk of CVD was higher in patients with CP (adjusted HR, 3.42) than those with AP (adjusted HR, 1.76) in the aforementioned study^5^. Increased risk of CVD in patients with CP were also reported in other studies, with a range of 1.27–1.453^25–27^. However, caution is needed in interpreting the results due to the heterogeneity of CP patients. Regarding research based on claims data such as our study, it is highly likely that true CP patients cannot be properly identified using only diagnosis codes. In our opinion, a more meticulous operational definition is required to perform the so-called big data study for CP. Accordingly, we recognized that it was difficult to set CP as a study outcome.

There are several limitations to our study. First, outcomes may vary depending on the etiology and severity of AP, which was not considered in our study. This is a somewhat intrinsic limitation of the NHIS database. Second, as mentioned above, participants that develop diabetes after AP are likely to be diagnosed with type 3c DM rather than T2DM. However, it was difficult to identify the number of such participants included in this study. Third, drugs administered to our participants were not considered. In diabetic patients, medications such as antithrombotic agents^28,29^ or metformin^5,30^ may affect the cardiovascular outcomes and mortality. Fourth, we did not evaluate other conditions such as angina, heart failure, or peripheral artery disease that belong to the category of CVD^31^. Lastly, there were statistically significant differences regarding several features of the two groups such as baseline characteristics including age, BMI, alcohol consumption, and smoking history which can directly affect CVD. In addition, the information about smoking and alcohol consumption obtained from surveys is likely to be underestimated. Such factors were difficult to manipulate by matching as there were many risk factors shared by AP and CVD. AP seems to play a certain role because we were able to obtain significant results even after we adjusted the confounding factors by the Cox proportional hazard ratio method. Nonetheless, the strength of our study is that we presented the risk of CVD and mortality, adjusting important confounding variables by long-term follow-up, according to AP history in a relatively uniform and large number of diabetic participants who had undergone health examinations.

## Conclusions

A significantly increased risk of CVD and mortality in diabetic patients with AP history was demonstrated in this large-scale nationwide cohort study. Regarding the risk management of diabetic patients, it can be crucial to check the history of AP as well as the major cardiovascular risk factors. To fully explain this increased risk, an accurate consideration of patients with pancreatogenic diabetes and detailed research on the long-term effects of AP on the cardiovascular system and metabolic components are warranted in the future.

## Data Availability

The derived data generated in the current study are available from the corresponding authors, upon reasonable request.

## Abbreviations

AP: Acute pancreatitis
BMI: Body mass index
CI: Confidence interval
CP: Chronic pancreatitis
CVD: Cardiovascular disease
HR: Hazard ratio
ICD-10: The international statistical classification of diseases and related health problems 10th revision
IL: Interleukin
MI: Myocardial infarction
NHIS: National health insurance service
T2DM: Type 2 diabetes mellitus
